# Evaluating Factors Influencing Knowledge-Sharing Behavior of Students in Online Problem-Based Learning

**DOI:** 10.3389/fpsyg.2021.691755

**Published:** 2021-06-25

**Authors:** Wei-Tsong Wang, Ying-Lien Lin

**Affiliations:** Department of Industrial and Information Management, National Cheng Kung University, Tainan, Taiwan

**Keywords:** social identification, interpersonal trust, relationship commitment, knowledge-sharing behavior, online problem-based learning, internship education

## Abstract

Adopting online problem-based learning (OPBL) to internship educational programs is an effective teaching method to stimulate self-directed and collaborative learning and knowledge-sharing behavior (KSB) of students. However, the OPBL collaboration experience is different from the traditional lecture-based learning experience for students. Integrating social identity theory and commitment-trust theory develops a formative research model that explains the KSB of students when using social media tools for the OPBL process. This process encourages social interaction and communication of students, in turn, facilitating the integration of collective intelligence or the creation, sharing, and exchange of knowledge. Data collected from 425 nursing students who studied at seven nursing colleges or medical universities in Taiwan were analyzed using the partial least squares (PLSs) technique. The results indicate that social identification is a crucial antecedent of KSB. Relationship quality plays a vital role in shaping the effects of interpersonal trust and relationship commitment (RC) on KSB during internship periods. The findings can contribute to theoretical discussions and enhance the effectiveness of KSB in the literature of internship and non-internship in the higher education field.

## Introduction

Developing problem-solving skills and higher-order thinking abilities of students is the objective of various educational institutions. Therefore, there have been prior studies on the adoption of the online problem-based learning (OPBL) approach in various educational contexts (e.g., Ding and Zhang, 2018; Car et al., 2019; Shimizu et al., 2019; Edelbring et al., 2020; Aslan and Duruhan, 2021). However, the dynamics of the use of social media tools (i.e., instant messaging services, social networking services, wiki systems, etc.) for facilitating OPBL in the context of internship educational programs in higher education institutes is under-addressed in the literature. For example, it is unclear whether the knowledge sharing behavior (KSB) of nursing students can be encouraged *via* social-media-supported (e.g., LINE, WeChat, and Facebook) collaborative learning activities that implement the OPBL teaching approach, especially in the context of nursing internship educational programs. On the other hand, OPBL is an effective self-directed and collaborative learning strategy that offers flexibility and opportunities for participation and discussion of students, encourages active learning, and promotes critical reasoning skills in various collaborative learning activities (Choi et al., 2014; Chu et al., 2019; Dolmans, 2019). In general, abilities of students are improved when problem-solving activities are conducted to encourage debates or discussion with peers, which can help them determine the best information/knowledge, fitting their needs to solve ill-structured and complicated real-world problems (e.g., Chang et al., 2012; Peng et al., 2021).

In the OPBL context, social media tools are often used as supportive learning tools to obtain the required knowledge and new information to promote collaborative learning, communication, or sharing of knowledge (Eid and Nuhu, 2011; Manca, 2020). Researchers suggested that individuals are likely to share their knowledge with the members of OPBL when they have a good interaction or relationship quality in a group (Ho et al., 2012; Tsai et al., 2014). Hosseini (2011) also argued that knowledge creation has four primary processes: socialization (e.g., unfreezing interactions, trust-building), externalization (e.g., sharing and constructing knowledge, critical thinking), combination (e.g., collaborative decision-making, group decision), and internalization (e.g., in-depth discussions, real-world applications). These processes provide students in an OPBL group with richer cognitive experiences or learning resources to help them cope with challenges and problems during internship periods. In the OPBL context, students may require more collaboration and KSB on patient care or problem-solving. Therefore, using social media tools can help them gain more field expertise and skills from peers, help them integrate collective intelligence to improve their professional skills, and deliver a better nursing service in the future (Liu et al., 2020). In general, both internship and non-internship educational programs are designed in an organized way to expand the professional knowledge and skills of students, provide them with growth opportunities, and assist them in building professional confidence in real-world practices. Research indicated that integrating an OPBL teaching strategy into the goals of a program is efficient for students (Edelbring et al., 2020).

Previous studies that compare the effectiveness of OPBL with that of face-to-face PBL have revealed that the learning efficiency or outcome of students is inconsistent in different PBL contexts (Erickson et al., 2020). Some studies suggest that social identity theory and commitment-trust theory are considered useful theoretical perspectives to investigate KSB in virtual groups (Hashim and Tan, 2015; Liou et al., 2016; Tsai and Hung, 2019). The two theories provide important insights into how interpersonal interaction and the relationship quality of an OPBL group influence the KSB of students (Lee et al., 2014). Individuals with high social identity levels are a critical determinant that drives their KSB in a group (Liou et al., 2016; Liao, 2017; Liu et al., 2021).

According to commitment-trust theory (Morgan and Hunt, 1994), maintaining strong relationships with group members will influence their sharing knowledge and values in an OPBL group. Then, they trust and commit to each other. Previous research has highlighted that interpersonal trust and relationship commitment (RC) in the interaction with the other party is crucial (e.g., Liou et al., 2016; Kim et al., 2020). The two elements are important determinants of relationship quality in a collaborative learning environment (Tseng and Kuo, 2014). They can help strengthen the loyalty of a member to an OPBL group, enable the development of interdependency among members, and discourage potentially unhelpful behaviors. In such case, KSB can be regarded as an outcome of the relational exchange or a knowledge transfer. Therefore, having reliable relational quality is a significant predictor in stimulating knowledge sharing exchange in OPBL activities.

To conclude, individuals can utilize social media tools to share knowledge when participating in discussions or training sessions/activities (Eid and Nuhu, 2011; Chu et al., 2019). It is the objective that nursing students learn the necessary knowledge and skills to support them in taking good care of patients in the workplace. Therefore, nursing internship educational programs emphasize social interaction, communication, and knowledge sharing to help students develop professional skills. This study integrates social identity theory and commitment-trust theory as the essential theoretical foundation to examine the key factors that influence the students’ KSB. This study is significant because few studies have integrated theoretical perspectives to investigate KSB in OPBL in nursing internship educational programs. Therefore, the research question (RQ) is as follows:

RQ: How does social identification of students influence the levels of relationship commitment and interpersonal trust in small group discussions using OPBL groups, which, in turn, affects their KSB?

## Literature Review and Hypothesis Development

Problem-solving skills are one of the critical capabilities that students of higher educational institutions have to acquire *via* educational programs. The development of this capability is highly dependent on the level of the effectiveness of knowledge acquisition anytime and anywhere. Prior studies on the adoption of the OPBL approach in various educational contexts have identified and examined a number of important factors, such as active learning and improving knowledge (Car et al., 2019; Shimizu et al., 2019), learning outcomes (de Jong et al., 2013; Ding and Zhang, 2018), motivation or cognitive load (Edelbring et al., 2020; Larmuseau et al., 2020), interaction or communication (Kim and Shin, 2014), and problem-solving skills (Chang et al., 2012; Aslan and Duruhan, 2021). Nevertheless, those prior studies exhibit inconsistent results regarding the effects of those factors on learning outcomes, attitudes, or behaviors of students, and they did not address those issues in the context of internship educational programs. Research indicates that interpersonal communications and interactions of students, and their perceived personal identity as members of internship programs may enhance of their expertise and skills, or result in positive attitudes toward learning groups to which they belong (Inceoglu et al., 2018). Therefore, examining the relationships among social identification, relationship quality, and KSB of students in the context of internship educational programs that adopt the OPBL approach can significantly contribute to the OPBL literature.

Since students often encounter complicated, multidisciplinary, and interdisciplinary problems in their classroom or field learning activities, the adoption of OPBL is beneficial. This is because OPBL is a student-centered teaching method that allows students to effectively reflect on their experiences and solve problems through collaboration. Therefore, using the OPBL teaching approach can promote the retention and transfer of knowledge learned through the dimension of the cognitive process of students (i.e., students autonomously acquire knowledge) rather than knowledge dimension (i.e., assisting the instructor to transfer knowledge to students). In this study, the nature of the problem is more related to knowledge acquisition through the cognitive process (analysis, synthesis, and evaluation) of students rather than through instructions (knowledge, comprehension, and application) of teachers (Budwig and Alexander, 2020). Additionally, the focal research topic of this study is related to problem-based learning, which promotes problem-solving and the accumulation of valuable experience and professional abilities of students.

### Social Identity Theory

Prior studies have widely applied social identity theory to research behaviors of computer-mediated communication (Postmes et al., 1998; Tsai and Bagozzi, 2014; Kim et al., 2019; Yilmaz, 2019). From the perspective of knowledge sharing, research indicated that social identification increases the online attachment motivation of students in virtual groups, which concentrates on stickiness intention (Yen, 2016) and collaborative participation (Cheung and Lee, 2010; Lee et al., 2014; Shen et al., 2014). However, existing studies have not yet fully explored how social identification would influence the KSB of students in OPBL groups. Social identity theory (Tajfel and Turner, 1979; O’Reilly and Chatman, 1986; Postmes et al., 1998) can help us understand intragroup phenomena and the learning process of an OPBL group. It explains how the attitudes of individuals define themselves in terms of in-group or out-group members. Ashforth and Mael (1989) argued that identification is assessing social categories or a social boundary relevant to a stable relationship and binding an individual to an intragroup. Social identification is the key for individuals to perceive themselves as a part of an intragroup when their relationship with other group members is based on cohesion, attachment, and reciprocity (Zuo et al., 2018). Members are willing to commit themselves to the benefits of the group, value the shared norms, and accept the obligations of the group to maintain their long-term benefits (Ashforth and Mael, 1989; Cheung and Lee, 2010; Liao, 2017). Therefore, the notion of belonging, similarities, and intimacy with others in a virtual OPBL group is critical in determining the social identification of individuals (Tsai and Bagozzi, 2014).

Some studies on virtual group identification have suggested that individuals who have a strong sense of belonging with their groups are likely to regard their goals as individual goals (Hsu et al., 2012; Yen, 2016). Based on this argument, it can be inferred that a high level of social identification encourages students to interact or share experiences, opinions, and expertise with peers, which can promote knowledge-sharing and other in-role or extra-role behaviors in an OPBL group (Yilmaz, 2019; Berger-Estilita et al., 2020). Previous studies have suggested that the two complementary social identity elements, namely, cognitive and affective, should be considered when investigating its formation and influences (Bergami and Bagozzi, 2000; Chou and Hsu, 2018). Cognitive identification is defined as the cognitive awareness that an individual is a member of the OPBL group. Affective identification refers to individuals’ positive feelings about an OPBL group, satisfying members’ important identity similarity (i.e., self-definitional requirements). This theory provides various fundamental identification criteria and behaviors (e.g., Ho et al., 2012; Lee et al., 2014; Liou et al., 2016).

### Commitment-Trust Theory

Commitment-trust theory (Morgan and Hunt, 1994) emphasizes the significance of developing long-term social relationships among individuals, groups, or organizations in business environments. Researchers have adopted commitment-trust theory to investigate online relationships (e.g., Morgan and Hunt, 1994; Shen et al., 2014; Liou et al., 2016) and continuance intention regarding knowledge-sharing virtual groups (Hashim and Tan, 2015). A learning group comprises individuals with similar experiences, concerns, or needs to achieve their goals, such as knowledge or information sharing and individual performance (de Jong et al., 2013), but there have been scarce studies that adopt this theory for investigating KSB in OPBL groups. Interpersonal trust, regarded as the dependability and reliability of relationship partners, is confirmed to encourage KSB in a group directly and indirectly through commitment (Chang et al., 2015). Additionally, knowledge-sharing is critical in advancing the problem-solving abilities of students and thus becomes a vital factor in collaborative learning in an OPBL group.

Since individuals are somehow unpredictable and uncontrollable by other people, collaborating with peers to solve problems tends to be a challenge in an OPBL group. In this study, interpersonal trust is defined as the extent to which students believe their peers will act in their interest. In addition, an interpersonal trust includes ability, integrity, and benevolence. Ability is viewed as an evaluation of the skills of OPBL members and competency in a learning group, which is related to captivating the professional knowledge of one; integrity refers to the reputation of honesty and truthfulness of the OPBL members; and lastly, benevolence is defined as the notion of reciprocal loyalty between an individual and an OPBL group, which would satisfy the expectations of behavior.

### Research Model and Hypothesis Development

**Figure 1:**
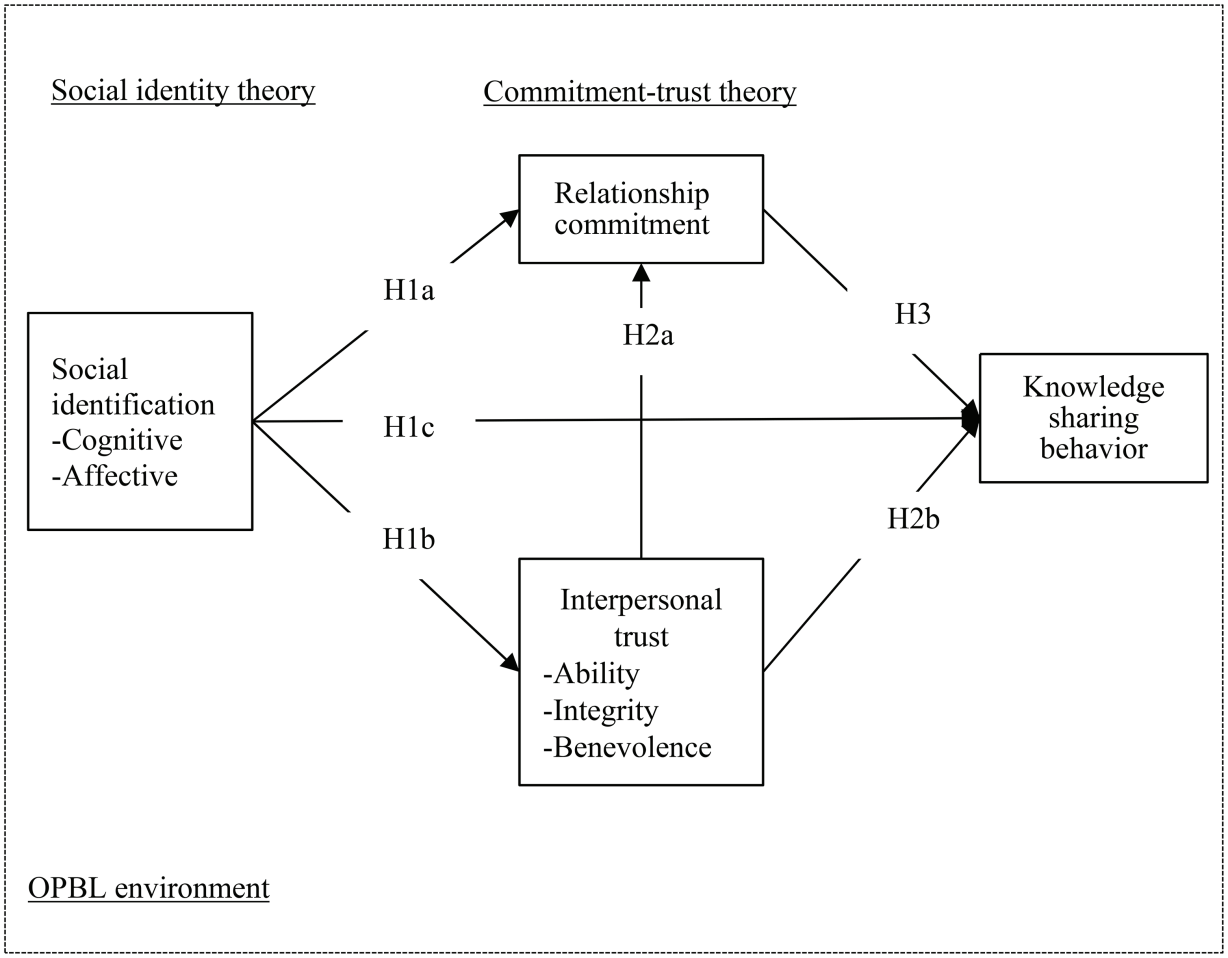
Social identification on KSB in an online problem-based learning (OPBL) environment.

This study mainly focuses on the KSB of students in OPBL activities using the LINE app to support collaborative learning in nursing internship educational programs. KSB refers to the degree to which nursing students share their experiences, professional knowledge, ideas, and answers to OPBL group questions. Figure 1 shows the research framework.

#### Social Identification

Identities of individuals with a particular objective in a group are characterized by a strong belief in accepting the values and goals of the group. As a result, they are willing to put extra effort into their group (Tsai and Bagozzi, 2014; Hamilton et al., 2018). Furthermore, strong beliefs and interactive experiences benefit students in building interpersonal trust and yielding their cognitive and affective reactions within a group (Cheung and Lee, 2010; Chou and Hsu, 2018). In other words, students with a high level of social identification can stimulate their trust in other members, and thus, intrinsically reduce the uncertainties of contribution to the group in any form (Ho et al., 2012; Kim et al., 2012; Rather, 2018). Therefore, we propose the following hypothesis:

*H1a*: Social identification of students is positively related to their interpersonal trust.

A high level of social identification leads individuals to perceive greater homogeneity with others, display their adherence to the norms of a group, and strengthen their loyalty to the members in an OPBL group (Ashforth and Mael, 1989). Social identification can maintain their relationship within the group and promote their commitment to an OPBL group (Bergami and Bagozzi, 2000; Hashim and Tan, 2015). Enhancing group identification of students is necessary to develop their intrinsic interests and reciprocate, motivate, and increase their sense of belongingness and willingness in sharing their interests, feelings, and opinions with their peers (Wilkins et al., 2016; Liao, 2017). Research has suggested that individuals with a strong social identification will be more committed to their relationship with others in an OPBL group (Tajfel and Turner, 1979; Kim et al., 2012; Rather, 2018). Therefore, we propose the following hypothesis:

*H1b*: Social identification of students is positively related to their relationship commitment.

Previous studies have indicated that both social identification and KSB or the quality of knowledge-sharing depict a strong linkage between an individual and members based on interaction frequency, friendship, and group task (Kim et al., 2019). Therefore, interpersonal interactions are a set of behaviors, such as information/knowledge exchange for assisting peers, which would affect the KSB of students (Ho et al., 2012; Yen, 2016; Lin et al., 2020; Liu et al., 2021). Therefore, we develop the following hypothesis:

*H1c*: Social identification is positively related to their KSB.

#### Interpersonal Trust

Interpersonal trust is a critical factor in building a close exchange relationship or the reliability of an exchange partner (Morgan and Hunt, 1994). Interpersonal trust can be characterized as a representation of the level of overall psychological safety of a team. When the level of interpersonal trust in an OPBL group is high, the group members tend to worry less about the potential negative consequences of their actions. They will pay more attention and energy to their learning tasks. Interpersonal trust promotes relationship commitment when building relationships with others, because it relies on positive feelings and improves relationship efficiency (Shen et al., 2014; Kim et al., 2020). Research found that interpersonal trust significantly affects the behaviors or intentions of a virtual group concerning an online/website user *via* relationship commitment mediation (Gharib et al., 2017; Lin et al., 2020). Therefore, we propose the following hypothesis:

*H2a*: Interpersonal trust of students is positively related to their relationship commitment.

Regarding interpersonal trust, Chang et al. (2012) have obtained insights into KSB by eliminating the perception of risk and uncertainty. An OPBL group with high interpersonal trust does not hesitate to share all knowledge/information. Interpersonal trust reduces concerns of knowledge contributors regarding knowledge appropriateness and misuse and enables their willingness to share sensitive and proprietary knowledge (Hosseini, 2011; Tsai and Hung, 2019). Previous studies have found that interpersonal trust primarily deals with integrity, benevolence, and ability, which increase KSB (Tsai et al., 2014). Thus, we inferred that interpersonal trust is a critical factor in influencing the KSB of students and their willingness to stay with an OPBL group during internship periods. Therefore, we propose the following hypothesis:

*H2b*: Interpersonal trust of students is positively related to their KSB.

#### Relationship Commitment

Relationship commitment is an essential factor in maintaining relational continuity among group members. Committed individuals are more likely to devote themselves to and put effort into knowledge-sharing activities in a group (Hashim and Tan, 2015). It can be inferred that students with a strong sense of belonging in an OPBL group can enhance their social interaction and relationship commitment with other members. That enables them to sustain long-term relationships (Morgan and Hunt, 1994) and stimulate KSB (Tseng and Kuo, 2014; Naeem et al., 2019). Thus, having a strong relationship commitment with members is beneficial to an OPBL group. Therefore, we propose the following hypothesis:

H3: Relationship commitment of students is positively related to their KSB.

## Methodology

### Participants and Procedures

The research background is mainly based on the internship programs of nursing education in Taiwan. First, we reviewed previous studies and summarized their findings that motivate this study. Additionally, we used the semi-construct interview method (1–2 h for each interview with a particular course instructor) to increase the validity of the research. Seven instructors accepted the interviews of the authors and shared their experiences concerning OPBL information in more detail. Finally, we integrated the two theories and examined the effects of critical factors on KSB performing rigorous statistical procedures to aggregate the questionnaire survey results.

Based on the responses of the interviewees in nursing internship educational programs (6 months or longer), each student has to achieve at least six skill-based clinical courses in order to be eligible to take the licensing examination for nursing practitioners. Students are assigned to multiple small groups; each group has less than eight students. In this study, the qualification of those nursing students who enrolled in various clinical courses is consistent with the sample selection purpose of this study. It can be inferred that they have sufficient experience or knowledge to answer the questions.

Additionally, the participants of this study, as members of learning groups, have to collaborate with one another in order to accomplish a shared learning goal. They did not receive any additional rewards except for getting good grades in the internship programs. Since students who belonged to the same learning group were assigned to the same hospital to do their internships, it was highly necessary for them to collaborate with one another in order to jointly resolve the problems that they encountered at work as an effective team. Since the healthcare problems of some patients were urgent, these students had to work together effectively to resolve the problems in a timely manner in order to deliver high-quality nursing services.

Furthermore, even though the students are in a learning environment in which they competed with one another to get the recognition of their instructors in order to acquire good grades, they were likely to cooperate with peers to ensure that they would pass the evaluation of the internship programs. To be more specific, because all the students in the internship programs needed to pass these courses to get the qualification to take the national nurse practitioner certification examination, they were willing to put significant efforts into collaboration with fellow students as a team in order to make sure that everyone could make it to the national nurse practitioner certification examination. This would lead to their benevolent KSB with the members of the OPBL groups, regardless of the possibility of reciprocated KSB from peers, although they might hope for the best. Therefore, to better explain such selfless KSB, this study identified the construct of social identification as one of the key antecedents of KSB in the proposed research model (see Figure 1).

The data of this study were collected from students in nursing internship education programs. We invited nursing students to participate in the survey based on their experience in internship educational programs. These procedures have been undertaken with the consent of the instructors of the internship programs appropriately. If the participants preferred to complete the questionnaires online, they could fill out our survey questions on the website by clicking a hyperlink. We provided a short description on the first page before asking related questions. All the participants were voluntary in response to the questions, and they could withdraw their participation in this survey at any time. Additionally, all the respondents remained anonymous in this survey.

We eventually received 467 replies from students who studied at seven nursing colleges or medical universities, yielding a response rate of approximately 90.5%. Because of the reason that respondents offered systematic answers or failed to pass the examination of reverse questions that they were purposely included in the survey, 42 problematic questionnaires were excluded. A total of 425 valid responses were used for subsequent data analysis procedures, yielding an effective response rate of 82.36%.

### Instrument Development

The instruments of this study were obtained from different studies, and the wording of all questions has been slightly modified to fit the unique research context. Social identification was measured using six items (three items for cognitive and three items for affective), adopted from Tsai and Bagozzi (2014). Interpersonal trust was measured using nine items (three items for ability, three items for integrity, and three items for benevolence) assessed by Shen et al. (2014) and Mpinganjira (2018). Relationship commitment was measured using five items by Vatanasombut et al. (2008). Finally, eight items were used to assess KSB, adopted from Chai et al. (2011). Additionally, we used a seven-point Likert scale ranging from (1) strongly disagree to (7) strongly agree to measure all items.

The overall sample population was 86.35% female. Of the students, 91.76% experienced LINE app for more than 2 years. During the progress of the internship programs, 84.48% spent more than 2 hours per day using the Internet for their homework. Additionally, 81.18% of the students reported using the LINE app, 80% used e-Library, and 73.65% used Google Scholar to support their learning activities. We can, thus, consider these students to be active users of computer-mediated technology and networks. Moreover, the students who completed the clinical courses, 88.94% completed fundamental nursing, 70.12% completed medical-surgical nursing, 68% completed psychiatric mental health nursing, 66.82% completed maternal and neonatal nursing, 65.41% completed community health nursing, 64.94% completed pediatric nursing, and 37.88% completed additional clinical nursing practices (e.g., comprehensive, acute care, long-term, and critical care).

### Data Analysis Methods

Partial least square (PLS), a component-based structured equation modeling technique, was used in this study. PLS has not required the normality of data distribution. It is appropriate to predict critical drivers of KSB because the research model of this study includes four constructs (social identification and interpersonal trust as the higher-order formative constructs) that are complicated (Petter et al., 2007; Wetzels et al., 2009; Hair et al., 2010). We used SmartPLS 3.0 to check the validity and reliability of the measures for the constructs in the proposed research model and then examine the hypotheses based on 425 valid data of survey respondents. A first-order reflective construct was used to assess the confirmatory factor measurement model for validity and reliability. At the same time, its latent indicators can perfectly explain the second-order formative construct for content validity. Additionally, this study performed a bootstrapping procedure (duplicated 5,000 times) for significant tests of the research hypotheses.

## Results

### Measurement Validation

First-order reflective constructs were used in the measurement model for testing its reliability and validity, which was assessed in terms of convergent and discriminant validity (Hair et al., 2010). Additionally, the reliability of the measurement model was evaluated by all values of Cronbach’s alpha coefficients using the first-order reflective constructs. The results show that all values were larger than 0.7 (ranging from 0.82 to 0.92). The convergent validity was evaluated in this study (Hair et al., 2010), including three methods that are considered acceptable: (i) the factor loadings of the indicators must be statistically significant and greater than 0.7; (ii) item reliability coefficient is assessed using composite reliability (CR), with values of 0.7 or higher; and (iii) average variance extracted (AVE) is used to estimate convergent validity, with values of 0.5 or higher. Table 1 shows that all of the factor loadings were statistically significant and ranged from 0.74 to 0.92. Furthermore, all CR values ranged from 0.89 to 0.93, and all of the AVE values ranged from 0.64 to 0.82. The three measures exhibited adequate convergent validity. Furthermore, the discriminant validity of the measurement model is also presented. Table 2 shows that the diagonal elements presented by the square root of AVE are larger than all the values of the correlation coefficients, which reveals that the constructs were strongly associated more with their respective indicators than with the other constructs.

**Table 1:** Results of factor loading, CR, and AVE.

Construct	Indicator	Factor loading	Composite reliability (CR)	Average variance extracted (AVE)
Social identification-cognitive (SIC)	SIC1	0.87	0.89	0.74
	SIC2	0.90		
	SIC3	0.80		
Social identification-affective (SIA)	SIA1	0.89	0.93	0.82
	SIA2	0.90		
	SIA3	0.92		
Interpersonal trust-ability (TA)	ITA1	0.89	0.91	0.77
	ITA2	0.88		
	ITA3	0.88		
Interpersonal trust-integrity (TI)	ITI1	0.88	0.90	0.76
	ITI2	0.91		
	ITI3	0.81		
Interpersonal trust-benevolence (TB)	ITB1	0.86	0.91	0.78
	ITB2	0.92		
	ITB3	0.86		
Relationship commitment (RC)	RC1	0.82	0.90	0.65
	RC2	0.75		
	RC3	0.87		
	RC4	0.74		
	RC5	0.85		
Knowledge sharing behavior (KSB)	KSB1	0.80	0.93	0.64
	KSB2	0.79		
	KSB3	0.79		
	KSB4	0.83		
	KSB5	0.80		
	KSB6	0.79		
	KSB7	0.81		
	KSB8	0.79		

All factor loadings are significant at *p* < 0.001.

**Table 2:** Descriptive statistics, correlations, and Cronbach’s alpha.

Measure	Mean	SD	Cronbach’s alpha	1	2	3	4	5	6	7
1. Cognitive	4.60	0.99	0.82	**0.86**						
2. Affective	4.62	1.10	0.89	0.76	**0.90**					
3. Ability	5.14	0.89	0.85	0.59	0.64	**0.88**				
4. Benevolence	5.21	1.03	0.86	0.66	0.66	0.69	**0.88**			
5. Integrity	4.99	1.05	0.84	0.64	0.64	0.62	0.73	**0.87**		
6. Relationship commitment	4.66	1.05	0.86	0.57	0.64	0.63	0.61	0.53	**0.81**	
7. Knowledge sharing behavior	4.68	0.94	0.92	0.67	0.69	0.59	0.69	0.56	0.62	**0.80**

*N* = 425; the *p*-value of all correlations is <0.01. SD, standard deviation. The square root of the average variance extracted is on the diagonal and bold values, and the other matrix entries are correlations.

Subsequently, social identification and interpersonal trust were conceptualized as second-order formative constructs that were formed as the weighted sum of their first-order reflective constructs. Petter et al. (2007) suggested that principal component analysis weights are better than evaluating factor loadings of indicators entirely. Table 3 shows that all the weights were significant. Table 2 indicates that the correlations among all of the first-order indicators showed reflective constructs of social identification and interpersonal trust (ranging from 0.56 to 0.76) and suggested a high-degree correlation, specifically because the substantial collinearity between variables was not present (Hair et al., 2010). We further examined the variance inflation factor (VIF) of the indicators for social identification and interpersonal trust to avoid excessive multicollinearity, which raises doubts about the validity of the formative model. Table 3 indicates that all the VIFs were smaller than the cutoff value of 3.3 (Petter et al., 2007), confirming that high multicollinearity was not present. Likewise, the content validity of the first-order indicators of the formative constructs of social identification and interpersonal trust was assessed using the magnitude of error terms, and Table 3 shows that all the error terms were small (*p* < 0.01) while all of the indicators’ coefficients were significant. These results indicate that their first-order indicators well-described social identification and interpersonal trust (Petter et al., 2007).

**Table 3:** Weight and variance inflation factor (VIF) of the formative indicators.

Second-order construct	First-order first-construct	VIF	Standard error	Weight (*t*-value)
Social identification	Cognitive	2.34	0.01	0.52 (56.22)
	Affective	2.34	0.01	0.55 (58.25)
Interpersonal trust	Ability	1.99	0.01	0.37 (30.44)
	Benevolence	2.66	0.01	0.40 (38.13)
	Integrity	2.28	0.01	0.35 (35.56)

### Structural Model and Hypothesis Testing

**Figure 2:**
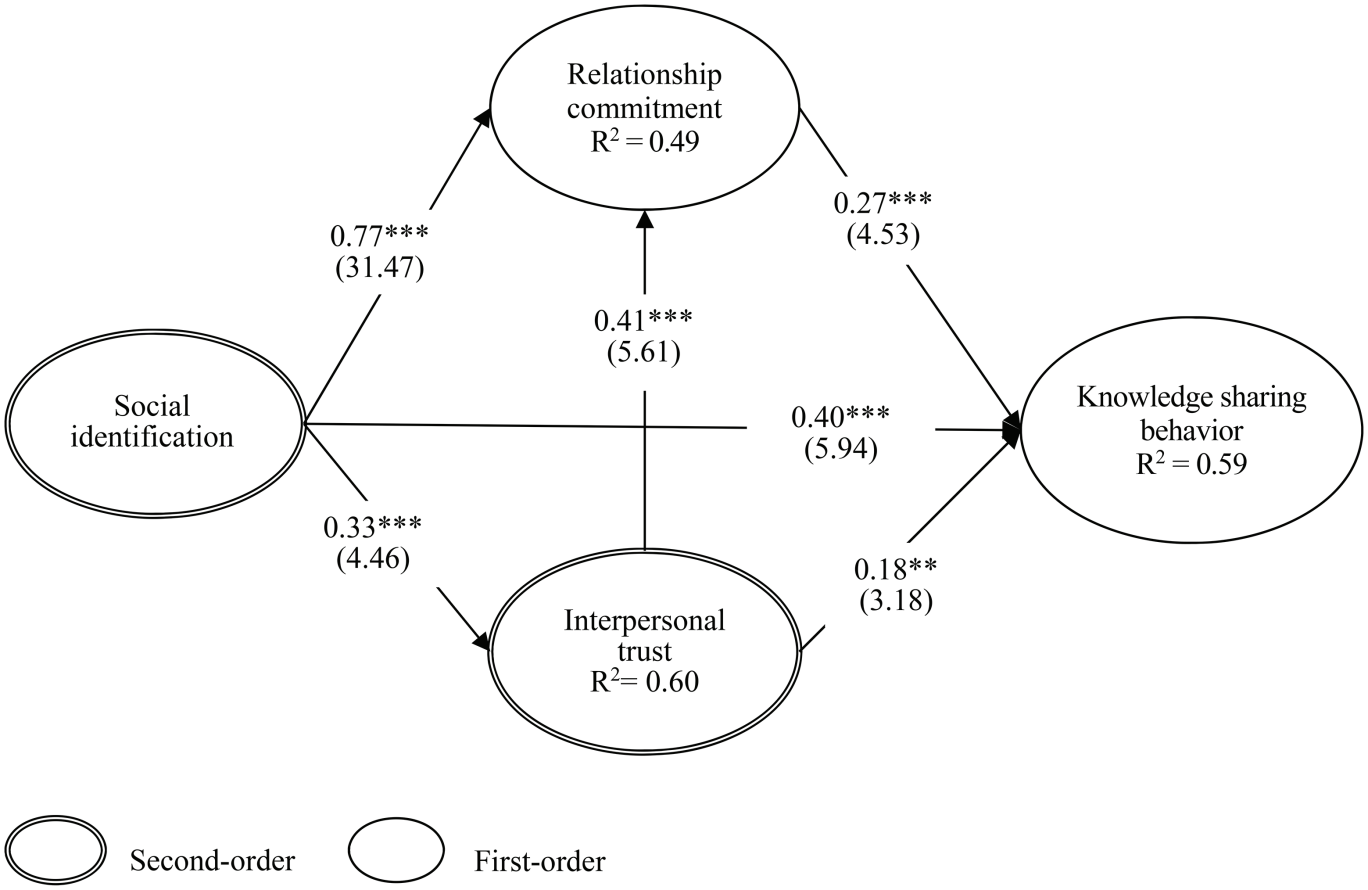
Results of path coefficient, *t*-value, and R square. ^***^p < 0.001; ^**^p < 0.01; Parentheses is *t*-value.

Using a bootstrapping technique by SmartPLS 3.0, we evaluated the hypotheses of the structural model. We used explained variances (R2) for the endogenous constructs to assess the structural model. The hypotheses of the proposed model are depicted in Figure 2, which presents the standardized path coefficients (β), *t* values, and R2 for each endogenous construct included in the proposed research model. Hypotheses H1a, H1b, and H1c are supported, indicating that social identification has a direct positive effect on interpersonal trust (*β* = 0.77, *t* = 31.47), relationship commitment (*β* = 0.33, *t* = 4.46), and KSB (*β* = 0.4, *t* = 5.94), respectively. Hypotheses H2a and H2b are both supported, indicating that interpersonal trust has a direct positive effect on relationship commitment (*β* = 0.41, *t* = 5.61) and KSB (*β* = 0.18, *t* = 3.18), respectively. Hypothesis H3 is supported, indicating that relationship commitment has a direct positive effect on KSB (*β* = 0.27, *t* = 4.53). The results indicate that social identification accounts for 60% of the variance of interpersonal trust. Additionally, social identification and interpersonal trust account for 49% of the variance of relationship commitment. Overall, all of the constructs that directly or indirectly influence KSB account for 59% of its variance.

## Discussion and Implications

The results of this study are as predicted. Social identification is positively related to relationship commitment (H1a), interpersonal trust (H1b), and KSB (H1c), consistent with prior research on social identification (Ho et al., 2012; Lee et al., 2014; Yilmaz, 2019; Liu et al., 2021). When the social identification of students in the OPBL group increases, the KSB of students in the OPBL activities is encouraged. Specifically, students need to sacrifice individual goals or time to achieve their OPBL group objectives. As individuals continue to move toward the OPBL group, their high identification results in more success in OPBL activities. Social identification provides a predictive and meaningful value of behaviors of members (relationship commitment and interpersonal trust) within OPBL groups.

Furthermore, interpersonal trust is positively associated with relationship commitment (H2a) and KSB (H2b), consistent with the results obtained in previous studies (Park and Chung, 2011; Ho et al., 2012; Naeem et al., 2019). Interpersonal trust and relationship commitment can contribute to developing favorable long-term interpersonal relationships, thus enhancing the cooperative behaviors of students. Cooperative behaviors may help eliminate barriers to the quality of KSB, increasing the trust of members in an OPBL group. Relationship quality, reflecting interpersonal trust and relationship commitment, can reduce the uncertainty of OPBL activities in a complex and dynamic workplace, maximize collaborative learning opportunities, and enhance stability and KSB among members.

Finally, relationship commitment is positively associated with KSB (H3), consistent with a previous study (Naeem et al., 2019). Relationship commitment plays a crucial role in helping students internalize norms and values of their community; and they are, thus, more likely to stay in their OPBL groups. A high level of relationship commitment leads to the willingness of students to actively communicate and share quantities of information/knowledge with other OPBL members. Besides, strong relationship commitment leads to a sense of belonging and group cohesion, which further enhances stability and strengthens relationships with other members for the necessities of professional growth of students in the nursing education field.

### Implications for Theory

The primary theoretical implication of this study is integrating the perspectives of relationship quality and social identity theory in the context of nursing internship programs that are conducted by implementing the OPBL approach and facilitating collaborative learning practice *via* the social media support. In the literature of online communities in the area of electronic commerce, relationship quality is recognized as a crucial mediator of the KSB of individuals (e.g., Liou et al., 2016; Ghahtarani et al., 2020; Lin et al., 2020). However, to the best of the knowledge of the authors, this viewpoint has not been validated in the context of higher education. Therefore, the findings of this study support the significance of the integration of social identity theory and commitment-trust theory in terms of examining the determinants of the KSB of students in internship and non-internship educational programs. We found that high social identification, relationship commitment, and interpersonal trust would encourage students to contribute their professional knowledge to OPBL activities, as did in some prior studies (e.g., Liou et al., 2016; Liu et al., 2021). Therefore, this study has addressed the research gap regarding the lack of development of a comprehensive theoretical view to underpin the main argument with research in prior studies of nursing education in higher educational institutes (Beccaria et al., 2018).

Furthermore, social identification is a crucial antecedent variable of interpersonal trust, relationship commitment, and KSB in the OPBL context (Xu et al., 2012; Wilkins et al., 2016). Social interaction of students is facilitated by their identification within the OPBL group that may generate adequate knowledge flows, interdependencies, and mutual respect among the OPBL members and, in turn, reduce stereotypes and enhance their professional development for individual growth (Berger-Estilita et al., 2020). The findings also reveal that quality factors of interpersonal interactions (i.e., relationship commitment and interpersonal trust) significantly affect the participation of students (i.e., learning by doing) in group activities and contribute to their KSB in their OPBL groups. This study extends the contributions of previous studies by integrating cognitive identification and affective identification, which are aggregated to be the construct of social identification in this study, to examine the effect of identification of students on KSB. In line with the argument of Hamilton et al. (2018), the adoption of the perspective of social identity theory in this study is appropriate, because this theory is effective in terms of comprehending the development of and variations in self-identity, attitudes, and values of students through social learning processes during their transitions between school environments and professional workplaces.

Traditionally, educators in eastern cultures, particularly in Taiwan, the educational systems of official examination and competitive learning atmosphere in Eastern cultures, which is different from those of educational systems in western cultures (Shimizu et al., 2019). Most students in Taiwan are not used to giving support, sharing experiences, or feelings with peers unless there is a known reason. Since students have different personalities, cultural beliefs, and learning styles, it would be difficult to ask them to contribute their knowledge, experiences, and opinions to an OPBL group (Shimizu et al., 2019). This study suggests that interpersonal trust enhances the willingness of students toward a vision of knowledge-sharing in an OPBL group. From the perspective of commitment-trust theory, interpersonal relationships promote collaborative learning in OPBL activities. Based on a strong psychological attachment to an OPBL group, relationship commitment is commonly characterized by active participation and KSB (Han et al., 2021).

### Implications for Practice

In a practical workplace, incorporating and leveraging the knowledge of OPBL groups is not an easy process. Social media tools allow students to provide professional connections between theory and practice and increase their professional competencies. Traditional teaching approaches may not be suitable for solving internship workplace problems when OPBL teaching methods have become more widespread. To succeed, OPBL groups should strengthen interactions, communications, and collaborations of their members to share or feedback their ideas, thinking, or opinions with peers by enhancing their sense of group membership (i.e., social identification) and their degrees of relationship commitment to and interpersonal trust in other group members, as suggested in prior studies (e.g., Tsai et al., 2014; Hashim and Tan, 2015). Therefore, in line with some prior studies, we suggest that the instructors of internship or non-internship courses may integrate social media tools into OPBL courses and provide students with guidance for fostering an atmosphere of collaborative learning. Such a collaborative-learning atmosphere can encourage students to perform active learning activities, evidence-based analogy, exploration, debates, and problem-solving, and be persistent and successful in learning challenging professional knowledge or skills (e.g., Liu et al., 2020; Peng et al., 2021).

During the progress of internship programs, students believe that the information/knowledge is credible and may gradually motivate their willingness to share their ideas or opinions and their commitment to other peers when they obtain useful and prompt feedback from their fellow peers in an OPBL group. In contrast, students who have a low interest in participating in OPBL activities may be encouraged to use the traditional learning method of rote memory to complete the clinical course that does not benefit the critical thinking and reasoning skills of students. The instructor can play an active role in collaborative learning to disseminate information while encouraging students to participate in professional communication in the OPBL context. Active participation in discussions may help students clarify false information, explain their ideas, and provide appropriate and accurate information to their peers. Additionally, instructors would need to respond to knowledge contributions (such as simple appreciations by posting a sticker) of students when students share their ideas, information, and opinions. Instructors are also required to regularly give comments for group discussions (e.g., cognitive skills for problem-solving), coach communication skills of students (e.g., conflict resolution), and encourage their exploration and collaboration. Finally, instructors may need to revise the traditional curriculum and combine various other materials, issues, and social media tools for designing content-laden curricula that are more appropriate for the needs of students of internship programs.

In an OPBL group, students with relationship commitment can readily connect and quickly exchange knowledge or information with peers beneficial to their communication and interaction expertise (Tsai and Hung, 2019). An OPBL group does not become overly dependent on a single student to share knowledge. Since the clinical environment is continuously changing, most students have to face professional skills shortages. Of more importance is that the participation and support of the instructor can increase the sense of belonging of students. We suggest that instructors guide students to reorganize their effective patient care strategies by utilizing social media tools to inspire students. Instructors can commit to students that the quantity and content of their knowledge contributions would not affect their grades, encouraging student participation, experience exchange, and KSB. Such context benefits students with more time to refine their learning and thinking with less shyness or nervousness.

### Limitations and Future Research Directions

This study obtains meaningful results; similarly, several limitations also are considered in this study. First, the environment of OPBL is different from that of general PBL teaching designs. This study focuses only on internship OPBL courses of nursing students who have to encounter real-world professional situations and deal with complicated and dynamic problems during the progress of their internship educational programs. Therefore, future studies can extend the contribution of this study by focusing on the examination of the research model of its extensions in various higher educational programs in other professional fields. Second, this study only considers the perspective of nursing students in the internship programs of the environment of OPBL communities. Factors related to the academic cultures, characteristics or styles of students, and perceptions of an instructor were not addressed in this study. We suggest that future research extends the proposed model by considering the factors mentioned above and use data collected from students in different professional areas or regions to investigate further how OPBL-based practices are used in an internship or non-internship educational programs. Third, because this study did not intend to investigate the differences in online and offline KSB of students, we did not collect data that would allow us to examine whether the effects of key factors identified on the KSB of students would be the same in online and offline situations. Future studies that focus on this issue may be conducted. Finally, most students lack critical thinking and communicative skills when they encounter real-world problems or patients. Therefore, the instructors should create an online collaborative learning environment to foster active participation and frequency of group interaction for filling in the knowledge gaps of students. Future research can investigate the role of instructors in sustaining the sense of belongingness of students in virtual communities that strengthen their interactions in OPBL activities.

## Data Availability Statement

The raw data supporting the conclusions of this article will be made available by the authors, without undue reservation.

## Ethics Statement

The authors’ University Governance Framework approved this study for Human Research Ethics (Number of Approval: HREC-109-088-2). The patients/participants provided their written informed consent to participate in this study.

## Author Contributions

All authors listed have made a substantial, direct and intellectual contribution to the work, and approved it for publication.

### Conflict of Interest

The authors declare that the research was conducted in the absence of any commercial or financial relationships that could be construed as a potential conflict of interest.
